# Specificity protein (Sp) transcription factors and metformin regulate expression of the long non-coding RNA HULC

**DOI:** 10.18632/oncotarget.4560

**Published:** 2015-07-06

**Authors:** Shruti U. Gandhy, Parisa Imanirad, Un-Ho Jin, Vijayalekshmi Nair, Eric Hedrick, Yating Cheng, J. Christopher Corton, KyoungHyun Kim, Stephen Safe

**Affiliations:** ^1^ Institute of Biosciences and Technology, Texas A&M Health Sciences Center, Houston, TX, USA; ^2^ Department of Veterinary Physiology and Pharmacology, Texas A&M University, College Station, TX, USA; ^3^ Integrated Systems Toxicology Division, US-EPA, MD B143-06, Research Triangle Park, NC, USA; ^4^ Department of Environmental Health, University of Cincinnati, Cincinnati, OH, USA

**Keywords:** HCC, Sp proteins, HULC, IncRNAs

## Abstract

Specificity protein 1 (Sp1) transcription factor (TF) regulates expression of long non-coding RNAs (lncRNAs) in hepatocellular carcinoma (HCC) cells. RNA interference (RNAi) studies showed that among several lncRNAs expressed in HepG2, SNU-449 and SK-Hep-1 cells, highly upregulated in liver cancer (HULC) was regulated not only by Sp1 but also Sp3 and Sp4 in the three cell lines. Knockdown of Sp transcription factors and HULC by RNAi showed that they play important roles in HCC cell proliferation, survival and migration. The relative contribution of Sp1, Sp3, Sp4 and HULC on these responses in HepG2, SNU-449 and SK-Hep-1 cells were cell context- and response-dependent. In the poorly differentiated SK-Hep-1 cells, knockdown of Sp1 or HULC resulted in genomic and morphological changes, indicating that Sp1 and Sp1-regulated HULC are important for maintaining the mesenchymal phenotype in this cell line. Genomic analysis showed an inverse correlation between expression of genes after knockdown of HULC and expression of those genes in liver tumors from patients. The antidiabetic drug metformin down-regulates Sp proteins in pancreatic cancer, and similar results including decreased HULC expression were observed in HepG2, SNU-449 and SK-Hep-1 cells treated with metformin, indicating that metformin and other antineoplastic agents that target Sp proteins may have clinical applications for HCC chemotherapy.

## INTRODUCTION

Hepatocellular carcinoma (HCC) is one of the leading causes of cancer deaths worldwide and over 500,000 new cases are diagnosed each year [1–3]. The incidence of HCC is highest in less developed countries where there is widespread hepatitis B virus (HBV) infection. The incidence of HCC in the United States has tripled over the past two decades and this is due, in part, to increased infection with hepatitis C virus (HCV) [1, 2]. Treatment for HCC is dependent on tumor stage and can include surgical resection, local ablation by radiofrequency treatment, transarterial chemobolization and radioembolization (TACE) [2] and the mechanism-based receptor tyrosine kinase (RTK) inhibitor sorafenib is also effective for treating patients with advanced HCC [4].

Recent studies show that several long non-coding RNAs (lncRNAs) are overexpressed in HCC and play important functional roles in liver cancer and therefore are also potential drug targets [5–16]. Highly upregulated in liver cancer (HULC) and high expression in HCC (HEIH) are two lncRNAs overexpressed in liver tumors. Both HULC and HEIH play a role in the proliferation of liver cancer cells. HULC is regulated by HBV X protein (HBX)-dependent activation of CREB which results in downregulation of the tumor suppressor gene p18 [7, 8]. The specificity protein 1 (Sp1) transcription factor regulates expression of HEIH in HepG2 and Huh7 cells [16], and genetic network analysis in HCC patients with HBV infection showed that Sp1 and peroxisome proliferator-activated receptor α regulated pathways are associated with early recurrence of HCC [17].

Sp1, Sp3 and Sp4 are overexpressed in many different cancer cell lines [18, 19] and high expression of Sp1 in tumors is a negative prognostic factor for breast, pancreatic, gastric, glioma, prostate and lung cancer patients' survival [20–26]. The relative expression of Sp transcription factors in tumors from HCC patients or in liver cancer cell lines has not been reported; however, there is indirect evidence that Sp1 and Sp-regulated genes are negative prognostic factors [16, 27, 28]. Several studies show that antineoplastic agents that include non-steroidal anti-inflammatory drugs (including COX-2 inhibitors), metformin, triterpenoids, curcumin and other natural products as well as ROS-inducing agents decreased expression of Sp1, Sp3, Sp4 and pro-oncogenic Sp-regulated genes [18, 29]. In this study, we show that HULC is an Sp-regulated gene and that the functional pro-oncogenic activities of HULC and Sp transcription factors are similar in liver cancer cells. Additionally, we show that HULC and Sp transcription factors are downregulated by the antidiabetic drug metformin.

## RESULTS

### Sp1, Sp3 and Sp4 transcription factors regulate HULC and other lncRNAs in HCC cells

**Figure 1:**
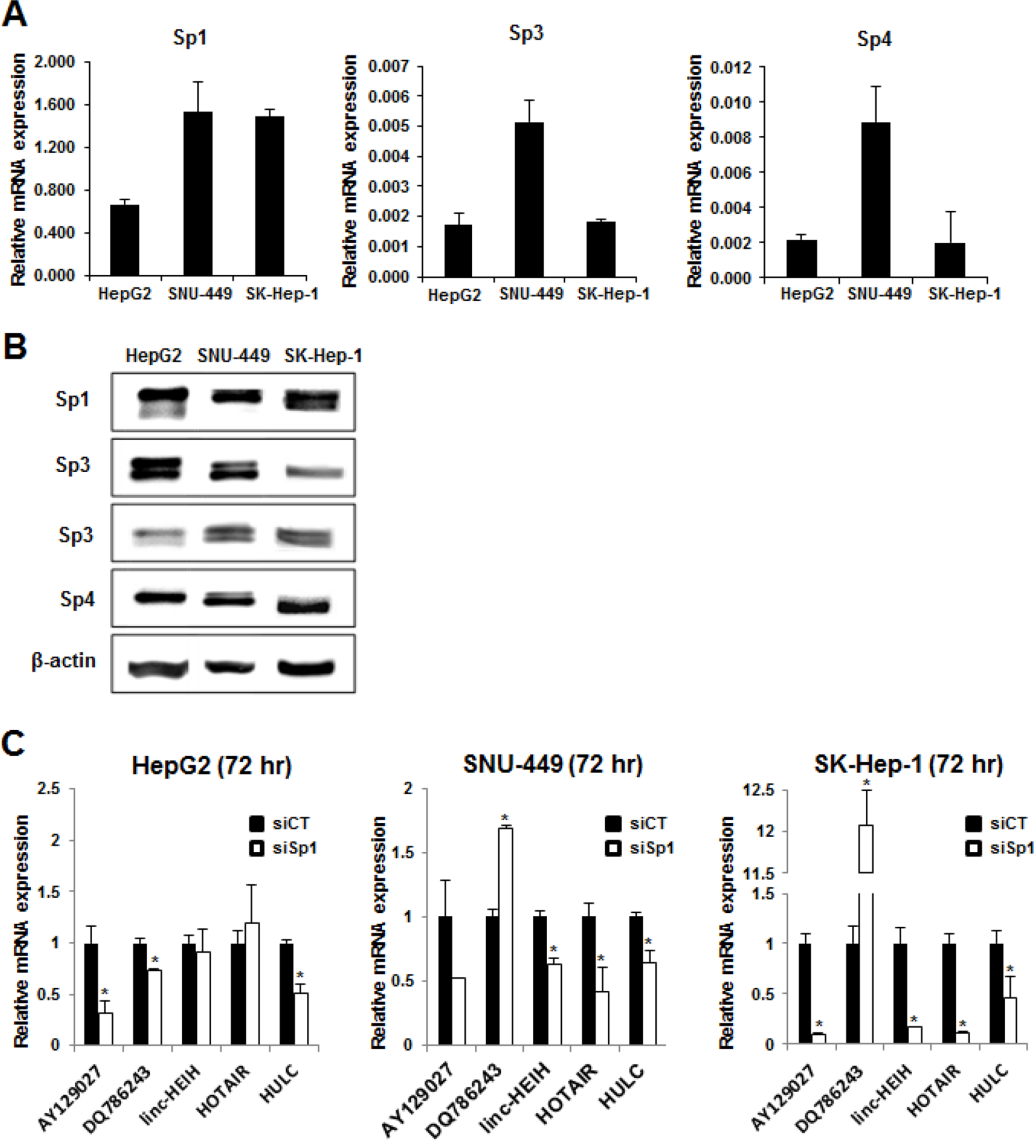
Sp protein expression and regulation of lncRNAs in HCC cells RNA **A.** and protein **B.** were isolated from HepG2, SNU-449 and SK-Hep-1 cells and analyzed for Sp1, Sp3 and Sp4 mRNA and protein expression by real time PCR and western blots, respectively as outlined in the Materials and Methods. **C.** Cells were transfected with siSp1 and lncRNA expression was determined by real time PCR as outlined in the Materials and Methods. Significant (*p* < 0.05) changes in expression compared to siCT are indicated (*).

The cell lines used in this study included HepG2 (slow growing, well differentiated), SNU-449 (modestly differentiated), and SK-Hep-1 (poorly differentiated) cells. Fig. 1A shows that Sp1, Sp3 and Sp4 mRNAs were expressed in all three cell lines with the highest levels of Sp1 mRNA in SNU-449 and SK-Hep-1 cells, and Sp3 and Sp4 mRNAs were more highly expressed in SNU-449 cells. Western blot analysis confirmed that Sp1, Sp3 and Sp4 proteins were also highly expressed in the three HCC cell lines. Using knockdown of Sp1 (siSp1) by RNA interference (RNAi) as a model, we initially investigated the effects of loss of Sp1 on expression of lncRNAs highly expressed in HCC cells, namely HULC, lncRNA-HEIH, AY129027, DQ786243 and HOTAIR [5] (Fig. 1C). Knockdown of Sp1 downregulated AY12907 in all three cell lines, and lncRNA DQ786243 was increasingly upregulated in the less differentiated cell lines. LncRNA-HEIH and HOTAIR were downregulated upon Sp1 knockdown in SNU-449 and SK-Hep-1 cells but their expression did not change in HepG2 cells. Interestingly, HULC was significantly and consistently downregulated upon Sp1 knockdown in all three HCC cell lines, suggesting that HULC is a Sp-regulated lncRNA in HCC cells.

**Figure 2:**
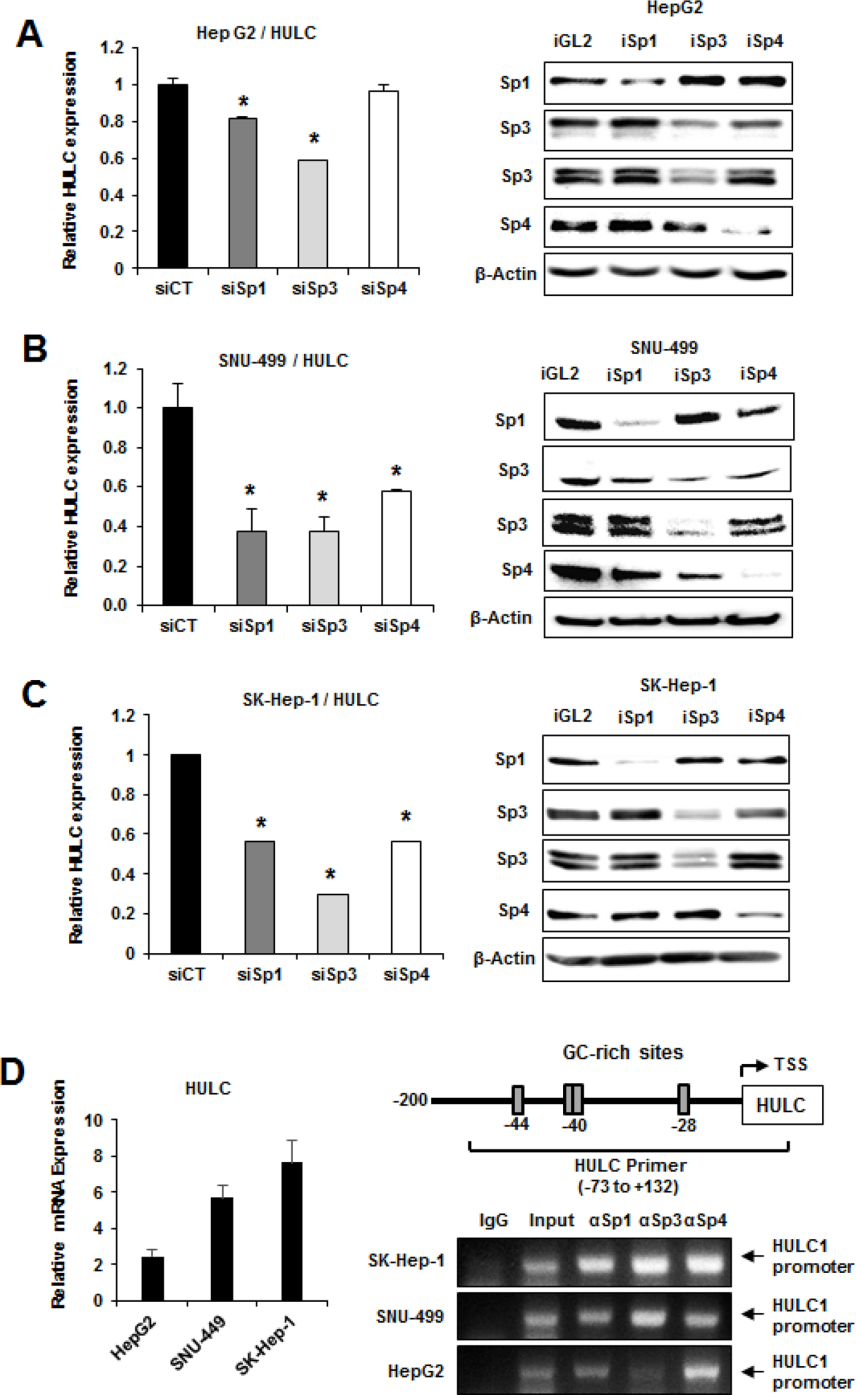
Sp transcription factors regulate HULC expression HepG2 **A.** SNU-449 **B.** and SK-Hep-1 **C.** cells were transfected with siSp1, siSp3 or siSp4, and HULC and Sp mRNA levels were determined by real time PCR as outlined in the Materials and Methods. Results are means ± SE for 3 replicate determinations, and significantly (*p* < 0.05) decreased gene expression is indicated (*). **D.** HULC expression in HCC cells was determined by real time PCR and binding of Sp1, Sp3 and Sp4 to the GC-rich HULC promoter was determined in a ChIP assay as outlined in the Materials and Methods and primers targeted the GC-rich region of the HULC promoter.

Regulation of HULC by Sp1, Sp3 and Sp4 in liver cancer cells was investigated by RNAi with oligonucleotides that targeted the individual Sp transcription factors. HULC expression in the more differentiated HepG2 cell was decreased approximately 20 and 40% after transfection of siSp1 and siSp3, respectively, but minimal effects were observed in cells transfected with siSp4 (Fig. 2A). In contrast, siSp1, siSp3 and siSp4 significantly decreased expression of HULC (>40–70%) in SNU-449 (Fig. 2B) and SK-Hep-1 (Fig. 2C) cells. Knockdown of Sp1, Sp3 and Sp4 in the HCC cell lines also decreased the protein expression encoded by several prototypical Sp-regulated genes including *BCL*-2, *SURVIVIN* and *EGFR* (Suppl. Fig. 1) as previously reported in other cancer cells [18, 29]. The HULC gene promoter has GC-rich sequences that bind Sp proteins (Fig. 2D). ChIP analysis showed that Sp1, Sp3 and Sp4 bound the GC-rich region of the HULC promoter in HepG2, SNU-449 and SK-Hep-1 cells and the observed binding was consistent with results showing that Sp transcription factors regulate HULC expression (Fig. 2C).

### Sp proteins and HULC regulate HCC cell proliferation, survival, migration/invasion

**Figure 3:**
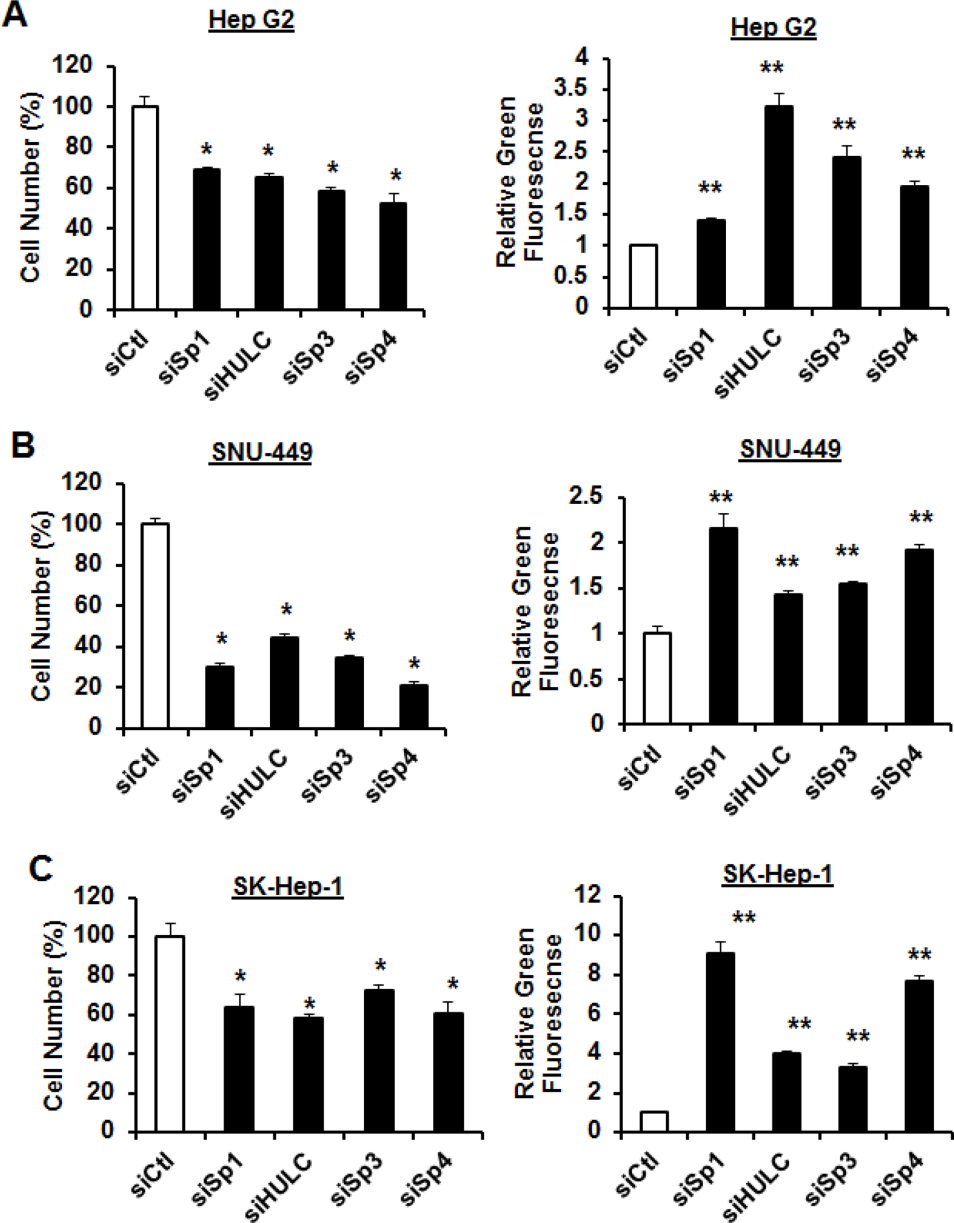
Knockdown of Sp1 and HULC decrease HCC cell proliferation and induce apoptosis siSp1, siSp3 and siSp4 effects on HepG2 **A.** SNU-449 **B.** and SK-Hep-1 **C.** cell proliferation and induction of Annexin V staining (marker of apoptosis) were determined as outlined in the Materials and Methods. Results are expressed as means ± SE for at least 3 replicate determinations, and significant (*p* < 0.05) inhibition of cell proliferation (*) or induction of green fluorescent Annexin V staining (**) are indicated. Transfection efficiencies for knockdown of HULC were >60% in all cell lines.

RNAi was also used to investigate the roles of Sp1, Sp3, Sp4 and HULC in regulating the proliferation and survival of HCC cells. We focused on HULC rather than AY12907 since knockdown of this lncRNA had minimal effects on cell proliferation (data not shown). Transfection of HepG2 (Fig. 3A), SNU-449 (Fig. 3B) and SK-Hep-1 (Fig. 3C) cells with siSp1, siSp3, siSp4 and siHULC inhibited HCC cell proliferation and induced apoptosis (Annexin V staining) and the magnitude of these effects was cell context-dependent. For example, Sp1 and Sp4 knockdown were the most effective inducers of Annexin V in SNU-449 and SK-Hep-1 cells, whereas Sp3 and Sp4 were the most active in HepG2 cells and siHULC induced Annexin V staining in all 3 cell lines. The percentage of cells with Annexin V staining was greater than 45–80%, 55–90% and 70–90% for HepG2, SNU-449 and SK-Hep-1 cells, respectively.

**Figure 4:**
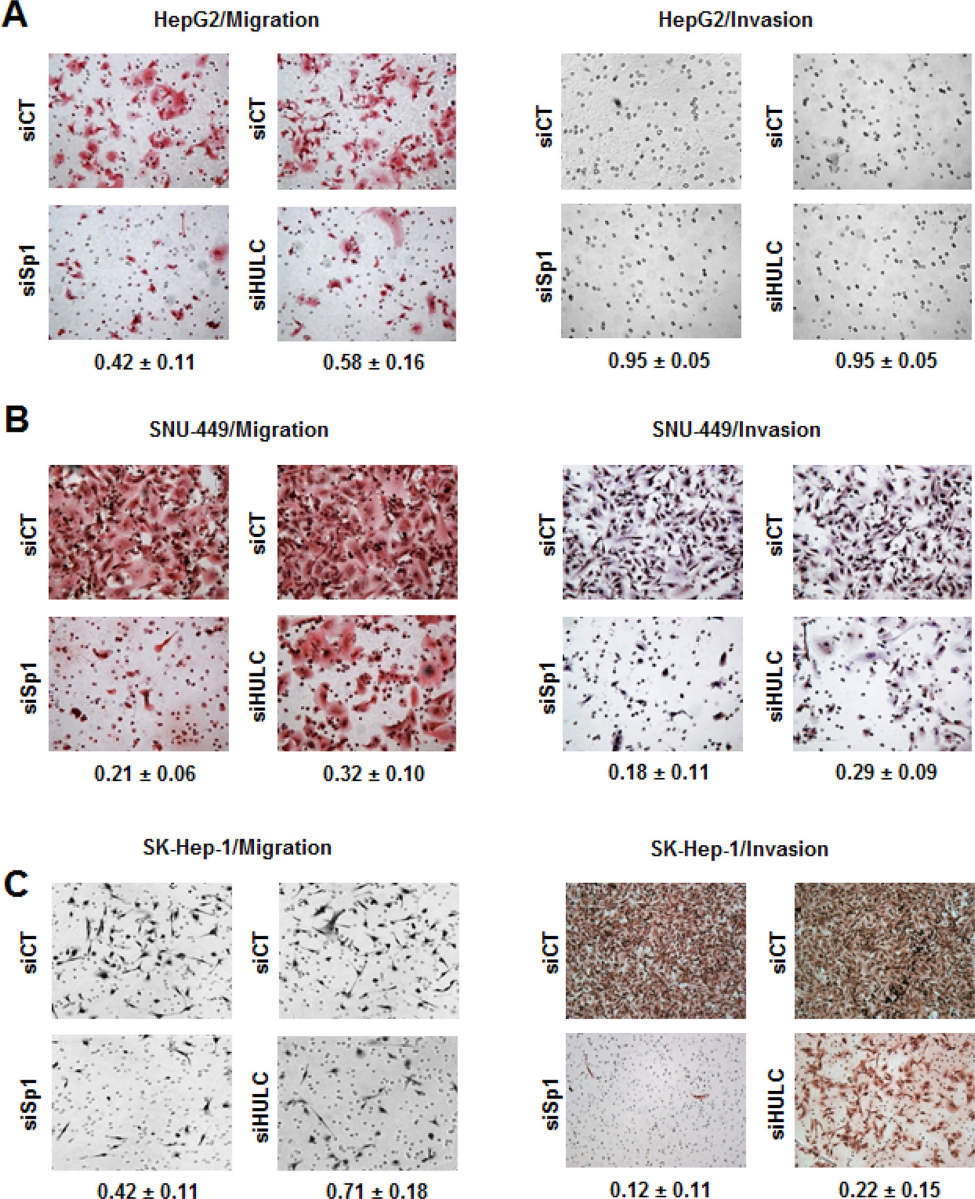
Knockdown of Sp1 and HULC inhibit HCC cancer cell migration and invasion HepG2 **A.** SNU-449 **B.** and SK-Hep-1 **C.** cells were transfected with siCT, siHULC or siSp1, and effects on cancer cell migration and invasion were determined as outlined in the Materials and Methods. Results are means ± SE for 3 replicate determinations, and values given in the Figures represent the fraction of cells migrating or invading compared to cell transfected with siCT (set at 1.0).

Using Sp1 knockdown as a model, HCC cells were transfected with siHULC or siSp1 and their effects on transwell migration and invasion of the three liver cancer cell lines were determined (Fig. 4). The results show that transfection of HepG2 (Fig. 4A), SNU-449 (Fig. 4B) and SK-Hep-1 (Fig. 4C) cells with siSp1 or siHULC significantly decreased cell migration in all the cell lines and decreased SNU-449 and SK-Hep-1 cell invasion in a Boyden chamber assay (Fig. 4B and 4C); effects were minimal for HepG2 cells (Fig. 4A) since invasion was not observed for this cell line. Responses observed in cells transfected with siSp1 were higher than siHULC, suggesting more significant contributions from other Sp-regulated genes.

**Figure 5:**
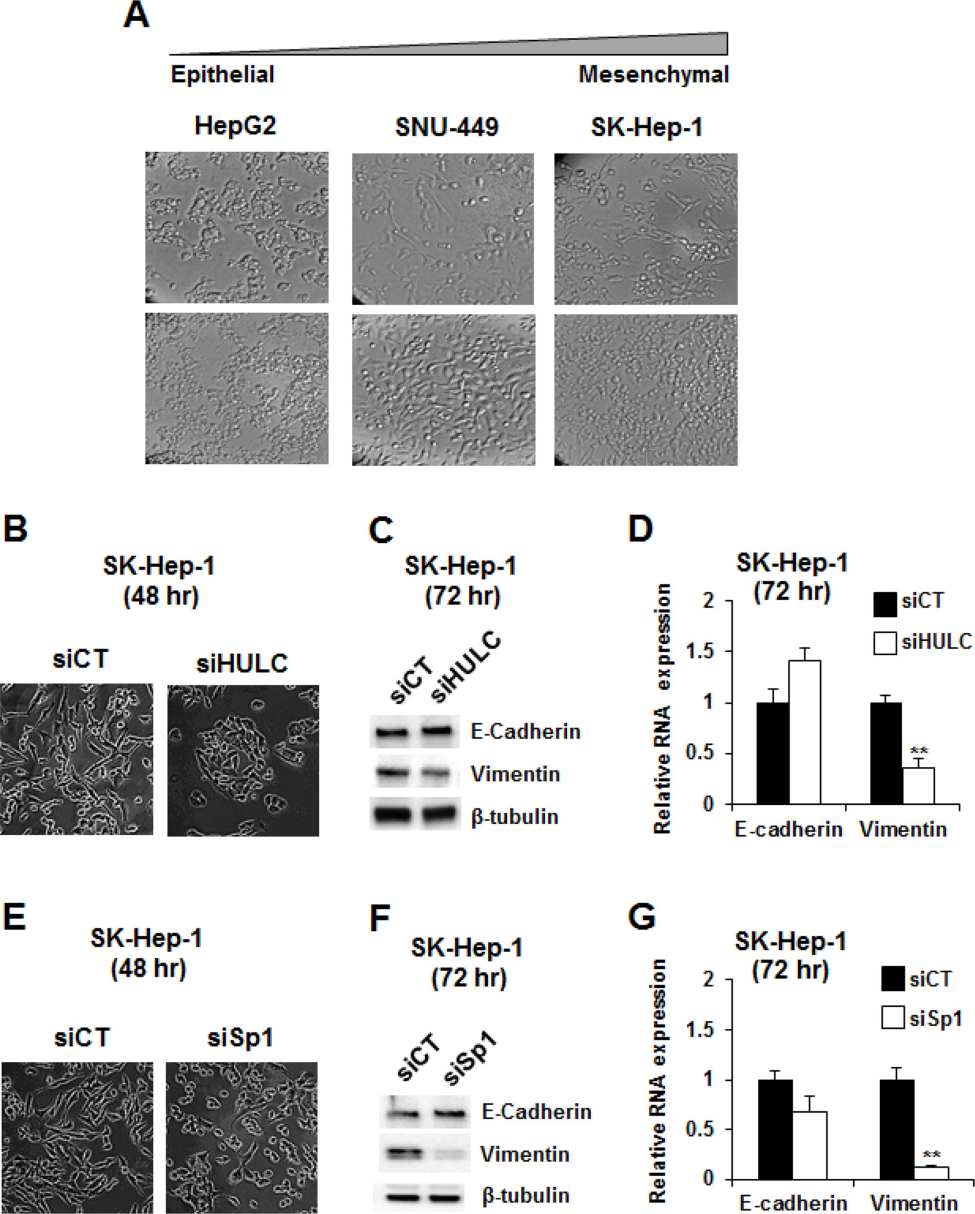
Modulation of epithelial-to-mesenchymal transition by knockdown of Sp1 and HULC **A.** The morphology of HepG2, SNU-449 and SK-Hep-1 cells was observed at both low and high confluencies using phase-contrast microscopy (10 × magnification). SK-Hep-1 cells were transfected with siHULC **B–D.** and siSp1 **E–G.** and changes in cell morphology, expression of E-cadherin and vimentin proteins and mRNA were determined by microscopy, western blots and real time PCR, respectively, as outlined in the Materials and Methods. Results (B and C) are expressed as means ± SE for 3 replicate determinations, and significantly (*p* > 0.05) decreased vimentin expression is indicated (**).

The cell morphologies of HepG2, SNU-449 and SK-Hep-1 varied according to their state of differentiation (Fig. 5A). HepG2 cells exhibited a typical epithelial phenotype with tightly clustered cuboidal cells, whereas the elongated and spindled-shaped phenotype of SK-Hep-1 cells resembled that of mesenchymal cells, and SNU-449 cells exhibited intermediate characteristics. These characteristics were consistent with a previous report showing that HepG2 cells exhibited higher expression of E-cadherin and lower expression of vimentin than SNU-449 cells [30]. We further investigated the role of HULC and Sp proteins in regulating epithelial to mesenchymal transition using the mesenchymal-like SK-Hep-1 cells as a model. Transfection of these cells with siHULC changed cell morphology, slightly increased E-cadherin mRNA and protein, and decreased expression of vimentin mRNA and protein (Fig. 5B–5D). Knockdown of Sp1 in SK-Hep-1 cells also changed cell morphology and had minimal effects on E-cadherin mRNA and protein but significantly decreased vimentin (mRNA and protein) (Fig. 5E–5G). Cell morphology in SK-Hep-1 cells after loss of HULC or Sp1 was more typical of epithelial cells, indicating that Sp1 and HULC played a critical role in maintaining the mesenchymal phenotype.

### HULC-regulated gene expression in cells and correlation with genes overexpressed in liver tumors

**Figure 6:**
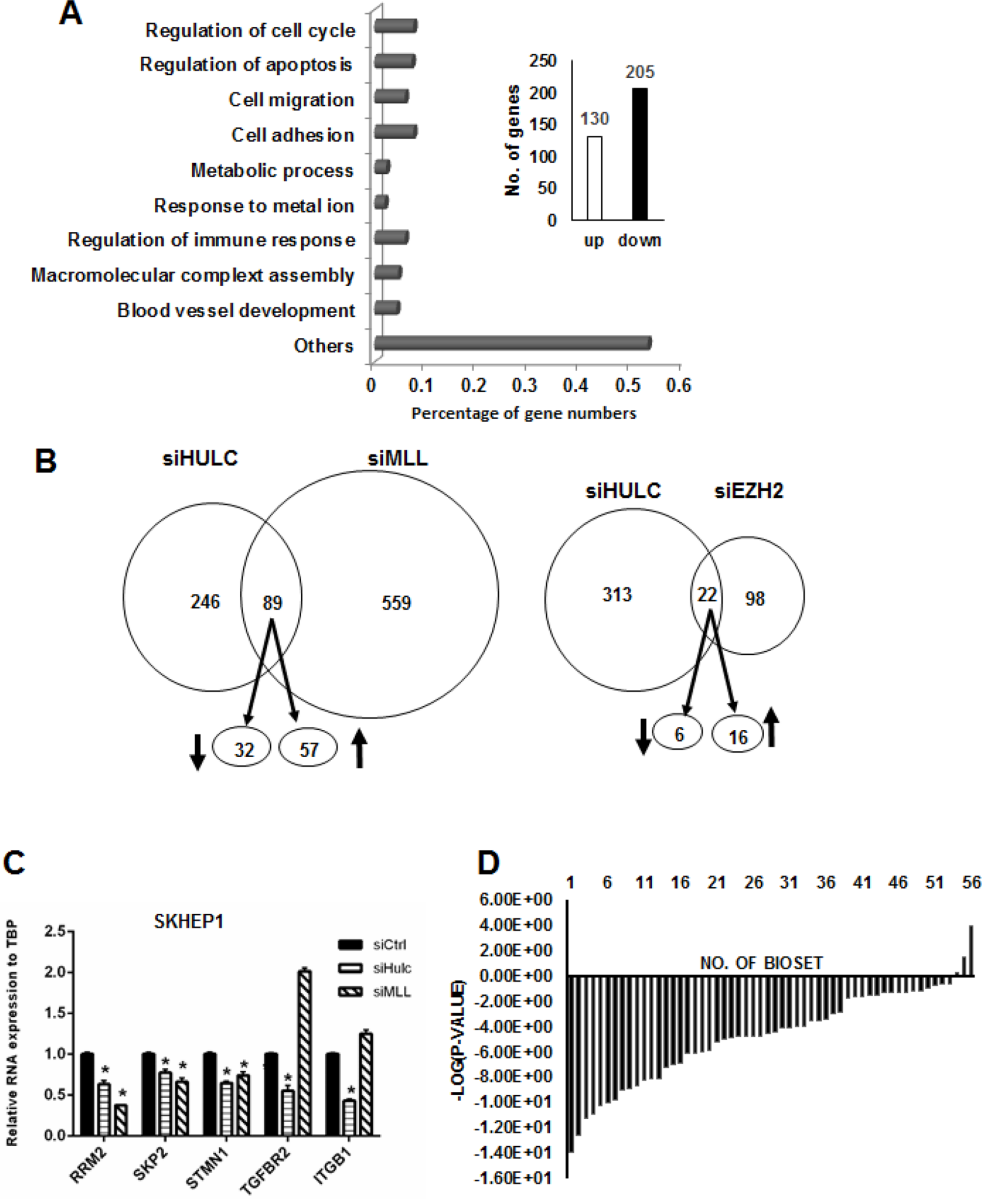
Regulation of genes by HULC and correlation with overexpressed genes in liver tumors **A.** Knockdown of HULC by RNAi in SK-Hep-1 cells modulated expression of 350 genes a determined using Illumina human HT12 v4 beadchip arrays and affected multiple cellular functions. **B.** Overlap of genes in SK-Hep-1 cells after combined knockdown of HULC and LMM1 and HULC and EZH2. **C.** Confirmation of selected HULC/MLL1 coregulated genes by knockdown and real time PCR. **D.** Significance of the overlap in the genes after HULC knockdown and in human liver tumors. Comparison between the HULC-regulated genes with genes altered in liver tumors vs. non-tumor tissues (56 biosets) was made using the Running Fishers test in NextBio.

Illumina human V.3 HT12 beadchip arrays were used to examine effects of transfection with siHULC on changes in gene expression in SK-Hep-1 cells. HULC knockdown resulted in the increase and decrease of 135 and 215 genes, respectively (Fig. 6A). Gene ontology analysis showed that HULC regulates expression of genes associated with cell growth/cell cycle expression, apoptosis, migration and several other functions. Many lncRNAs interact with chromatin modifying complexes to regulate gene expression and since mixed linkage leukemia 1 (MLL1) and enhancer of zeste homology 2 (EZH2) are both involved in hepatocarcinogenesis [31, 32] and also components of chromatin-modifying complexes, we compared the overlap of genes in SK-Hep-1 cells transfected with siHULC with siMLL1 and siEZH2. Fig. 6B illustrates the number of common genes coregulated by HULC/MLL1 and HULC/EZH2 after knockdown experiments (siHULC, siMLL1 and siEZH2) in SK-Hep-1 cells. The overlap in HULC/EZH2 coregulated genes was minimal, whereas HULC/MLL1 coregulated 32 and 57 common genes thatwere down- and upregulated, respectively (Fig. 6B) (Supplemental Table 1). Real time PCR was used in a separate experiment to demonstrate that individual knockdown of siHULC or siMLL1 decreased expression of RRM2, SKP2 and STM1 in SK-Hep-1 cells (Fig. 6C) and this pattern was also observed in the array data (Supplemental Table 2). The TGFBR2 and ITBG1 genes were decreased only by siHULC and this also corresponded to the array data. However, knockdown of MLL1 increased TGFBR2 and ITGB1 gene expression, suggesting that MLL-1 regulation of these genes may be indirect and do not involve coregulation by the MLL-1/HULC complex. Given that knockdown of HULC expression negatively affected the growth and survival of hepatocellular carcinoma cell lines (Fig. 3), we hypothesized that the expression of HULC-regulated genes would negatively correlate with expression of those genes in liver cancer. A total of 350 genes (≥1.5-fold) regulated by HULC knockdown were compared to the profiles of human liver tumors vs. normal liver in the NextBio database. Out of the total of 56 comparisons, all but three exhibited negative correlation to the HULC-regulated genes, i.e., those genes down-regulated by HULC knockdown were up-regulated in the liver tumors and those genes up-regulated by HULC knockdown were down-regulated in the tumors (Fig. 6D). More than half of these biosets (39 out of 56) exhibited statistically significant negative correlation to the HULC-regulated genes (alpha = 0.05 with a Benjamini-Hochberg multiple test correction).

### Metformin decreases expression of Sp transcription factors and HULC

**Figure 7:**
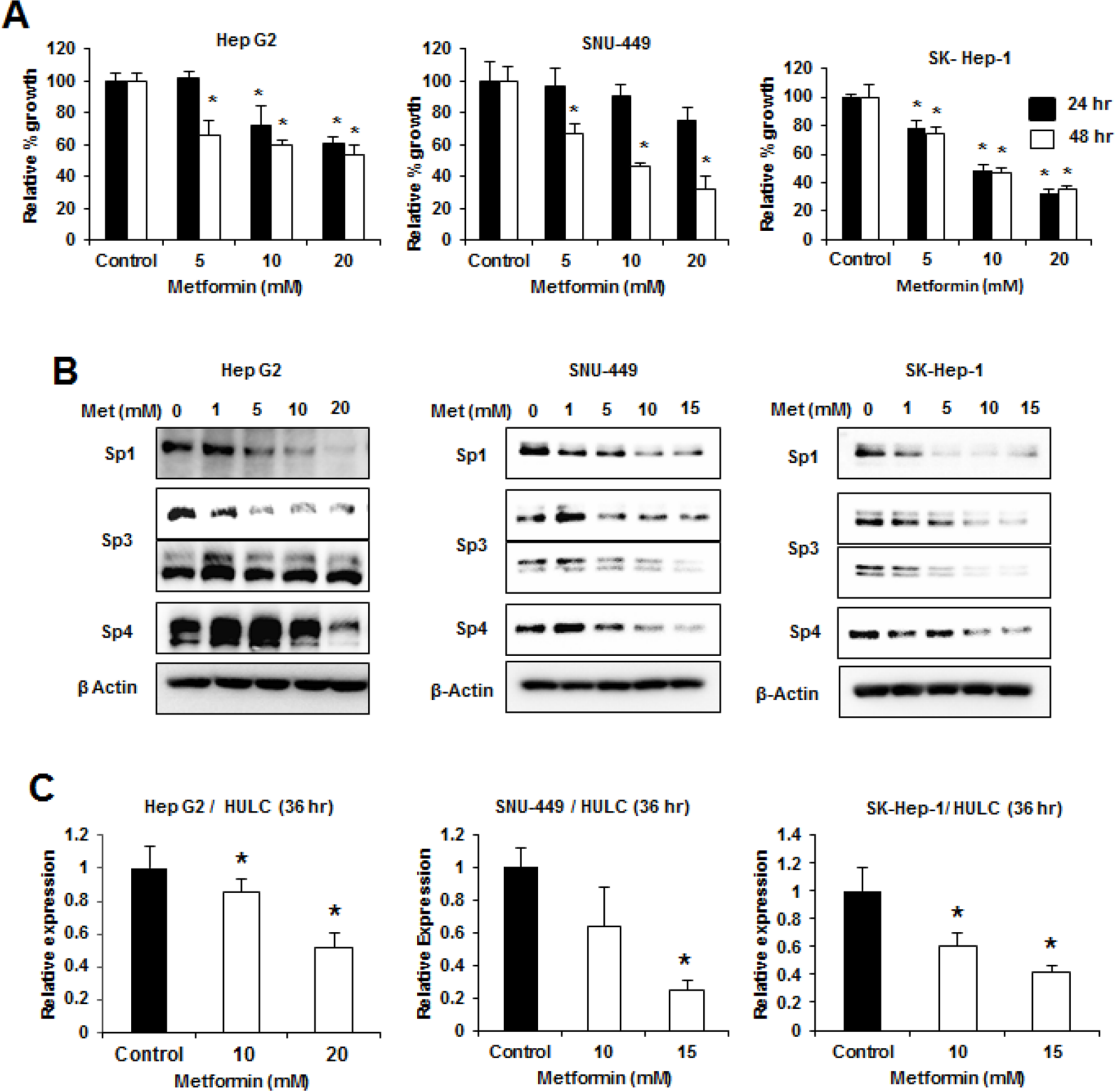
Metformin decreases growth, Sp proteins and HULC expression in HCC cells HCC cells were treated with DMSO (control) and 1–20 μM metformin for the indicated times, and effects on cell proliferation **A.** and expression of Sp1, Sp3 and Sp4 proteins **B.** were determined by cell counting and western blots, respectively, as outlined in the Materials and Methods. **C.** HCC cells were treated with DMSO, 10 or 15 mM metformin for 36 hours and expression of HULC (relative to DMSO treatment) was determined by real time PCR as outlined in the Materials and Methods. Results (A and C) are expressed as means ± SE for 3 replicate determinations and significantly (*p* < 0.05) decreased responses are indicated (*).

The antidiabetic drug metformin [33] decreases expression of Sp1, Sp3, Sp4 and Sp-regulated genes in pancreatic cancer cells [18] and metformin (0–20 mM) inhibited growth of HepG2, SNU-449 and SK-Hep-1 cells after treatment for 24 and 48 hr (Fig. 7A). Metformin also decreased expression of Sp1, Sp3 (high molecular weight), and Sp4 in HepG2 cells after treatment for 24 hr; however, the lower molecular weight Sp3 band was not affected, whereas in SNU-449 cells, the higher molecular weight Sp3 band was only slightly decreased (Fig. 7B). In contrast, Sp1, Sp3 (high and low molecular weight), and Sp4 were decreased in the less differentiated SK-Hep-1 cells after treatment with metformin for 24 hr (Fig. 7B) and after treatment for 48 hr, all the Sp transcription factors were decreased in the three cell lines (data not shown). Metformin also decreased expression of HULC in HepG2, SNU-449 and SK-Hep-1 cells (Fig. 7C), demonstrating that drugs targeting Sp transcription factors also target Sp-regulated lncRNAs in HCC cells. Metformin treatment represents a novel mechanistic approach for treating HCC patients that overexpress Sp transcription factors and HULC.

## DISCUSSION

Sp transcription factors are overexpressed in multiple tumors and cancer cell lines and there is evidence that Sp1 is a negative prognostic factor for patients with several different cancers including HCC [20–26]. Sp transcription factors are not only negative prognostic factors for patient survival but also exhibit pro-oncogenic functional activity as determined by knockdown studies in several cancer cell lines [18, 29]. For example, loss of Sp1 in fibromyosarcomas, gliomas, multiple myeloma, lung, pancreatic and colon cancer cells, and rhabdomyosarcomas results in one or more of inhibition of growth, cell cycle progression, survival, migration and invasion [29, 33–43]. The effects resulting from Sp1 knockdown are consistent with the parallel decrease in pro-oncogenic Sp-regulated genes associated with cancer cell survival (bcl-2, survivin), proliferation (EGFR, c-MET, cyclin D1), angiogenesis (VEGF and VEGFR1), inflammation (p65NFκB), and invasion (CXCR4 and MMP9) [18]. Sp3 and Sp4 regulate many of the same functions and genes described for Sp1; however, the role of Sp1, Sp3 and Sp4 are both gene- and cell context-specific.

In this study, we show that Sp1, Sp3 and Sp4 mRNA and protein are highly expressed in HepG2, SK-Hep-1 and SNU-449 HCC cell lines (Fig. 1A and 1B). It was previously reported that expression of lncRNA-HEIH was decreased after knockdown of Sp1 by RNAi in HepG2 and Huh7 cells [16] and therefore, we initially used RNAi to investigate the role of Sp1 in regulating a panel of lncRNAs identified in HCC cell lines. Surprisingly, we did not observe decreased expression of lncRNA-HEIH as previously reported [16] and this may be due to a temporal effect which is currently being investigated. Decreased expression of Sp1 differentially modulated expression of the lncRNAs in HepG2, SNU-449 and SK-Hep-1 cells and HULC was one of only two lncRNAs that were Sp1-regulated in all three cell lines (Fig. 1C). Moreover, we also observed that knockdown of Sp1 and Sp3 decreased HULC expression in HepG2, SNU-449 and SK-Hep-1 cells and siSp4 decreased HULC in the latter two cell lines (Fig. 2). Sp1, Sp3 and Sp4 also interacted with the GC-rich HULC promoter in HepG2, SNU-449 and SK-Hep-1 cells (Fig. 2D), further demonstrating a role for Sp transcription factors in regulating this lncRNA. Previous studies showed that PKA and CREB upregulate HULC expression and that insulin-like growth factor mRNA binding protein 1 (IGF2BP1) destabilizes HULC [9], whereas our results demonstrate that Sp transcription factors are important for basal expression of HULC in HCC cells.

Sp transcription factors play a role in the proliferation, survival, migration/invasion of several cancer cell lines [18] and we used RNAi to investigate the differential effects of Sp1, Sp3, Sp4 and HULC knockdown on HCC cell proliferation and survival. Loss of the transcription factors and HULC individually decreased cell proliferation and induced apoptosis in all three cell lines and SNU-449 and SK-Hep-1 cells were more sensitive than the more highly differentiated HepG2 cells. Knockdown of Sp1 and HULC also decreased migration and invasion of HCC cells (Fig. 4) and partially reversed the mesenchymal phenotype of SK-Hep-1 cells in which the mesenchymal marker vimentin was decreased (Fig. 5). We also further characterized the role of HULC in liver cancer by examining the modulation of gene expression after knockdown of HULC or knockdown of two important components of chromatin-modifying complexes (MLL1 and EZH2) (Fig. 6). Analysis of array data demonstrated the multifunctional roles of HULC which correlated with the functional effects of knockdown of HULC. Coregulation of genes by HULC and EZH2 or HULC and MLL1 was limited (Fig. 6C); however, there was significant negative correlation between HULC-regulated genes in SK-Hep-1 cells with genes regulated in hepatocellular carcinomas (Fig. 6D). Currently, we are further investigating diagnostic genes overexpressed in tumors and their coregulation by HULC and Sp transcription factors and other chromatin modifying complexes.

Results of this study demonstrate the importance of HULC and Sp transcription factors in HCC, suggesting that both HULC and Sp1 can be targeted by anticancer agents that are known to downregulate Sp transcription factors in other cancer cell lines and tumors [18, 35–40, 43]. Metformin is an antidiabetic drug that protects patients against pancreatic cancer and metformin downregulates Sp1, Sp3, Sp4 and pro-oncogenic Sp-regulated genes in pancreatic cancer cells and tumors [33]. Metformin also protects diabetic patients from development of HCC, and metformin regulated genes/pathways in HCC cell lines are similar to those reported in pancreatic and other cancer cell lines [44–47]. In this study, we show that metformin inhibits growth of HepG2, SNU-449 and SK-Hep-1 cells, and the growth inhibition is accompanied by downregulation of Sp transcription factors and Sp-regulated HULC expression (Fig. 7). Thus, the antineoplastic effects of metformin are due, in part, to downregulation of Sp proteins and HULC and we are currently investigating other Sp-regulated pathways in HCC cells that contribute to this response.

In summary, this study demonstrates that Sp1 and other Sp transcription factors are important for liver cancer cell growth, migration, survival, invasion and epithelial-mesenchymal-transition, and we demonstrate that HULC and other lncRNAs are Sp-regulated genes in HCC. Thus, drugs such as metformin that downregulate Sp transcription factors and Sp-regulated genes (e.g. HULC) may be clinically useful in drug combinations for treating HCC patients that overexpress Sp transcription factors. Moreover, serum levels of HULC [6, 10] may be a marker of treatment efficacy.

## MATERIALS AND METHODS

### Cell culture, reagents, and antibodies

HepG2, SNU-449, and SK-Hep-1 cells were obtained from American Type Culture Collection (Manassas, VA). Cells were cultured in Dulbecco's modified Eagle's Medium (DMEM) with High Glucose supplemented with 10% fetal bovine serum (FBS), 10 mL/L of 100× antibiotic/antimycotic solution (Sigma-Aldrich Co., St. Louis, MO). Cells were grown in 150-cm^2^ culture plates in an air/CO_2_ (95:5) atmosphere at 37°C. Antibodies were purchased as follows: Sp1, Sp3 and Sp4 (Millipore, Billerica, MA); E-cadherin (Cell Signaling Technology, Danvers, MA), vimentin (Sigma-Aldrich), β-tubulin (Santa Cruz Biotechnology, Santa Cruz, CA).

### Small interfering RNAs and transfection

Small interfering RNAs (siRNAs) targeting human HULC RNA (siHULC), Sp1 (siSp1), Sp3 (siSp3), and Sp4 (siSp4) were purchased from Sigma-Aldrich. Negative and Positive (Cell Death) Control siRNA were purchased from Qiagen. Transfection was carried out using Lipofectamine RNAiMAX (Invitrogen) according to manufacturer's protocol.

### Cell proliferation assay and annexin V staining

Cell proliferation and Annexin V staining was carried as previously described [29]. For knockdown experiments with siRNAs, effects of cell proliferation and Annexin V staining were determined after 72 hr.

### Quantitative real time PCR (qRT-PCR) and western blot analysis

Total RNA was isolated and analyzed by qRT-PCR as described [29]. Primers were purchased from Integrated DNA Technologies and the primers are summarized in Supplemental Table 2. Western blot analysis of whole cell lysates was determined as previously described [29].

### Transwell migration and invasion and chromatin immunoprecipitation (ChIP) assays

Migration and invasion assays were performed using 24-well transwell chamber with 8 μM pore size polycarbonate membrane (Corning Costar), and invasion assays were carried out under the same conditions with the exception of the transwell chamber used as described [29]. The ChIP assay was performed using ChIP-IT Express Magnetic Chromatin Immunoprecipitation kit (Active Motif, Carlsbad, CA) according to the manufacturer's protocol essentially as described [29].

### Gene analysis

Total RNA was extracted from Panc1 cells by using mirVana™ miRNA Isolation Labeling Kit (Ambion, Inc./LifeTechnologies, Grand Island, NY) and quantified by using Nanodrop ND-1000 spectrophotometer (NanoDrop Technology, Wilmington, DE). HumanHT-12 v4 expression beadchip arrays (Illumina, Inc., San Diego, CA) were used for determining changes in gene expression according to the manufacturer's protocol and microarray data were normalized using the quantile normalization method in the Linear Models for Microarray Data (LIMMA) package in the R language (http://www.r-project.org). Differentially expressed genes were identified using *a* > 1.5-fold change cut off. Gene ontology enrichment analysis was carried out using David Functional Annotation Resources 6.7 (http://david.abcc.ncifcrf.gov/). A rank-based nonparametric analysis strategy called the Running Fisher's algorithm and implemented within the NextBio database (http://www.nextbio.com/) environment was used to identify gene expression comparisons (biosets) which have statistically significant positive or negative correlation to the genes regulated by siHULC in SK-Hep-1 cells. The Running Fisher's algorithm computes statistical significance of similarity between ranked fold-change values of two gene lists [48]. After exporting the analysis, the list of correlated biosets were filtered to identify those that examined gene expression changes in liver tumors.

### Statistical analysis

Statistical significance of differences between the treatment groups was determined using the Student's *t* test, and levels of probability were noted.

## SUPPLEMENTARY FIGURE AND TABLES



## Abbreviations

ChIP: chromatin immunoprecipitation
DMEM: Dulbecco's modified Eagle's medium
FBS: fetal bovine serum
HBV: hepatitis B virus
HBX: HBV X protein
HCC: hepatocellular carcinoma
HULC: highly upregulated in liver cancer
IGFBP1: insulin-like growth factor binding protein 1
lncRNA: long non-coding RNA
NSAIDs: non-steroidal anti-inflammatory drugs
PVDF: polyvinylidene difluoride membrane
qRT-PCR: quantitative real time PCR
RNAi: RNA interference
RTK: receptor tyrosine kinase
siRNA: small inhibitory RNA
Sp1: specificity protein 1
TACE: transarterial chemobolization and radioembolization
TBP: TATA binding protein
TBST: Tris-buffered saline with Tween
VEGF: vascular endothelial growth factor
VEGFR: vascular endothelial growth factor receptor
