# Supplementary material for: Specificity protein (Sp) transcription factors and metformin regulate expression of the long non-coding RNA HULC
Source: Oncotarget. 2015 Jul 6;6(28):26359–72. doi: 10.18632/oncotarget.4560 (PMC4694907; doi:10.18632/oncotarget.4560)
Supplement: Supplementary file 1 [file oncotarget-06-26359-s001.pdf]

SUPPLEMENTARY FIGURE AND TABLES

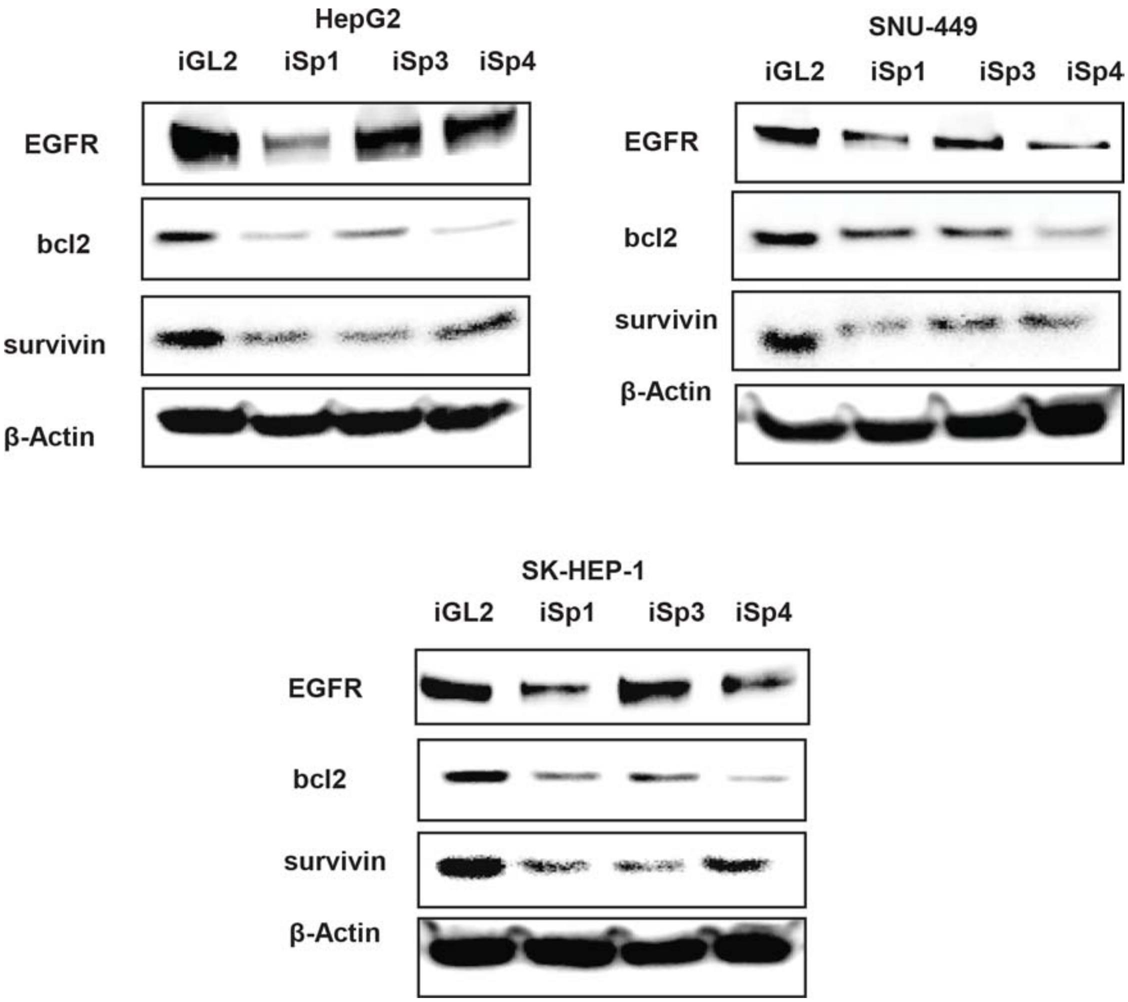

Supplementary Figure S1: Sp knockdown decreases Sp-regulated genes. Cells were transfected with siSp1, siSp3 and siSp4, and whole cell lysates were analyzed by western blots as outline in the Materials and Methods.

Supplementary Table S1. Summary of genes downregulated in SK-HEP-1 cells transfected with siHULC or siMLL1

Gene name	Gene definition	siHULC fold change	siMLL1 fold change
PAK2	p21 protein (Cdc42/Rac)-activated kinase 2	0.44	0.64
SLC27A2	solute carrier family 27 (fatty acid transporter), member 2	0.46	0.53
WDR34	WD repeat domain 34	0.48	0.58
FAM111A	family with sequence similarity 111, member A	0.5	0.63
RPL39L	ribosomal protein L39-like	0.52	0.6
CXADR	coxsackie virus and adenovirus receptor pseudogene 2; coxsackie virus and adenovirus receptor	0.53	0.63
C19orf33	chromosome 19 open reading frame 33	0.55	0.57
ELOVL2	elongation of very long chain fatty acids (FEN1/Elo2, SUR4/Elo3, yeast)-like 2	0.57	0.62
LOC399942	hypothetical gene supported by AF081484; NM_006082; tubulin, alpha 1b	0.59	0.64
TUBA1A	tubulin, alpha 1a	0.59	0.66
RTN3	reticulon 3	0.6	0.56
DTL	denticleless homolog (Drosophila)	0.61	0.59
ERCC6L	excision repair cross-complementing rodent repair deficiency, complementation group 6-like	0.61	0.62
RRM2	ribonucleotide reductase M2 polypeptide	0.61	0.57
SKP2	S-phase kinase-associated protein 2 (p45)	0.61	0.58
LOC730316	similar to Nuclear envelope pore membrane protein POM 121	0.62	0.57
PLS1	plastin 1 (I isoform)	0.62	0.66
STMN1	stathmin 1	0.62	0.63
H2AFX	H2A histone family, member X	0.63	0.54
ROD1	ROD1 regulator of differentiation 1 (S. pombe)	0.63	0.49
CDKN3	cyclin-dependent kinase inhibitor 3	0.64	0.37
F3	coagulation factor III (thromboplastin, tissue factor)	0.65	0.66
MCM10	minichromosome maintenance complex component 10	0.65	0.6
MNS1	meiosis-specific nuclear structural 1	0.65	0.56
PRIM2A	PRIM2 primase, DNA, polypeptide 2 (58kDa)	0.65	0.56
CAST	calpastatin	0.66	0.65
CDCA7	cell division cycle associated 7	0.66	0.64
EZH2	enhancer of zeste homolog 2 (Drosophila)	0.66	0.66
GTF3C2	general transcription factor IIIC, polypeptide 2, beta 110kDa	0.66	0.59
RGS4	regulator of G-protein signaling 4	0.66	0.61
SPAG5	sperm associated antigen 5	0.66	0.62
TMPO	thymopoietin	0.66	0.59

Supplementary Table S2. Primers used for total RNA isolation and quantitative real time PCR analysis

Purchased from Integrated DNA Technologies	
Sp1 (forward):	5′-TCA CCT GCG GGC ACA CTT-3′
Sp1 (reverse):	5′-CCG AAC GTG TGA AGC GTT-3′
TBP (forward):	5′-TGCACAGGAGCCAAGAGTGAA-3′
TBP (reverse):	5′-CACATCACAGCTCCCCACCA-3′
HULC (forward):	5′-atctgcaagccaggaagagtc-3′
HULC (reverse):	5′-cttgcttgatgctttggtctgt-3′
AY129027 (forward):	5′-TGG AAT GAA GGC AGG GCT AA-3′
AY129027 (reverse):	5′-CTC TTC TTA CAA GGA CGC CAG T-3′
lncRNA-HEIH (forward):	5′-CCTCTTGTGCCCCTTTCTT-3′
lncRNA-HEIH (reverse):	5′-ATGGCTTCTCGCATCCTAT-3′
DQ786243 (forward):	5′-TCTGGGAGGCTGAAGTTGT-3′
DQ786243 (reverse):	5′-GTGAAGGGATTTGGAGGG-3′
HOTAIR (forward):	5′-GGTAGAAAAAGCAACCACGAAGC-3′
HOTAIR (reverse):	5′-ACATAAACCTCTGTCTGTGAGTGCC-3′
Vimentin (forward):	5′-GCCATCAACACCGAGTTGAAG-3′
Vimentin (reverse):	5′-CGATGTAGTTGGCGAAGCG-3′
E-cadherin (forward):	5′-ACAGACATGAGCCACTGCACC-3′
E-cadherin (reverse):	5′-CGATGGAGCGAAACTGTCTCTTA-3′
Snail (forward):	5′-GACTACCGCTGCTCCATTCCA-3′
Snail (reverse):	5′-TCCT-CTTCATCACTAATGGGGCTTT-3′
Slug (forward):	5′-CCATTCCACGCCCAGCTA-3′
Slug (reverse):	5′-GGACTCACTCGCCCCAAA-3′
ZEB1 (forward):	5′-GAAAATGAGCAAAACCATGATCCTA-3′
ZEB1 (reverse):	5′-CAGGTGCCTCAGGAAAAATGA-3′
ZEB2 (forward):	5′-TTCCATTGCTGTGGG-CCTT-3′
ZEB2 (reverse):	5′-TTGTGGGAGGGTTACTGTTGG-3′
**Purchased from Sigma**	
RRM2 (forward)	5′-GACACAAGGCATCGTTTCAA-3′
RRM2 (reverse)	5′-TCTATGGCTTCCAAATTGCC -3′
SKP2 (forward)	5′-GAAGGGAGTCCCATGAAACA -3′
SKP2 (reverse)	5′-GCTGAAGAGCAAAGGGAGTG -3′
STMN1 (forward)	5′-TCTCGTGCTCTCGTTTCTCA-3′
STMN1 (reverse)	5′-TTCCCTGGAGGAAATTCAGA -3′
TGFBR2 (forward)	5′-TCTGGTTGTCACAGGTGGAA-3′
TGFBR2 (reverse)	5′-GCACGTTCAGAAGTCGGTTA -3′
ITGB1 (forward)	5′-CAGTCCAATCCAGAAAATTGG-3′
ITGB1 (reverse)	5′-GAGTCGCGGAACAGCAG-3′
